# Supplementary material for: Adaptations for stealth in the wing-like flippers of a large ichthyosaur
Source: Nature. 2025 Jul 16;644(8078):976–83. doi: 10.1038/s41586-025-09271-w (PMC12390834; doi:10.1038/s41586-025-09271-w)
Supplement: Supplementary file 1 — Part A. Depositional environment. Part B. Specimen details and taxonomic assignment. Part C. Ontogenetic assessment. Part D. Osteoderms. Part E. Reconstructing the front flipper of Temnodontosaurus. Part F. Notes on analyses and experiments. Part G. Notes on the taphonomy. Part H. Function of the flippers in Temnodontosaurus. Part I. Large eyes and adaptations for stealth in ichthyosaurs. Part J. References cited in the Supplementary Information. [file 41586_2025_9271_MOESM1_ESM.docx]

**Table of contents**

Part A. Depositional environment

Part B. Specimen details and taxonomic assignment

Part C. Ontogenetic assessment

Part D. Osteoderms

Part E. Reconstructing the front flipper of *Temnodontosaurus*

Part F. Notes on analyses and experiments

Part G. Notes on the taphonomy

Part H. Function of the flippers in *Temnodontosaurus*

Part I. Large eyes and adaptations for stealth in ichthyosaurs

Part J. References cited in the Supplementary Information

**Part A. Depositional environment**

During the Early Jurassic, much of what is today Central Europe comprised an archipelago of islands surrounded by the European Epicontinental Sea, which included several basins^1,2^. The sediments of the Posidonia Shale (Posidonienschiefer Formation) formed within the Southwestern German Basin (SWGB) between 30 and 35° N palaeolatitude during the latest Pliensbachian and Toarcian^1–4^. Within this rock unit, the Posidonia Shale Konservat-Lagerstätte is renowned for its exceptionally preserved fossils^4^.

Stratigraphically, the lower Toarcian part of the Posidonia Shale is sub-divided into three ammonite zones, ranging from the *Dactylioceras* *tenuicostatum* zone at the bottom, followed by the *Harpoceras* *serpentinum* zone, with the *Hildoceras bifrons* zone at the top^1,4,5^. The sedimentary sequence is composed mainly of marls, marly clays and dark limestones^1^, with the oil shales and fossilised animal soft tissues occurring principally within an interval that stretches from the upper part of the *D*. *tenuicostatum* zone to the middle part of the *H*. *serpentinum* zone^2,6^.

Several depositional models have been proposed to explain the formation of the Posidonia Shale^2^. Of these, a combined synthesis of sedimentological, geochemical, palaeoecological, and particulate organic matter data supports the so called ‘silled-basin’ model^2–4^. It postulates that limited open ocean water circulation reached the SWGB as the result of a regression during the late Pliensbachian, followed by only a minor transgression into the basin, whereby shallow-marine conditions with restricted circulation were created^2–4^. These conditions were interrupted by periods of improved water circulation due to seasonal fluctuations governed by summer monsoons and dry winters, allowing for the sporadic colonisation of the seafloor by benthic organisms^1,7^.

While it has long been thought that anoxic conditions in the bottom water were responsible for the exceptional preservation of animal soft parts^1,2,8,9^, both Muscente *et al*.^4^ and De La Garza *et al*.^10^ recently hypothesised that these remnants were phosphatised (the dominant mode of fossilisation in the Posidonia Shale) in a low oxygen environment (rather than under complete anoxia).

While marine vertebrates occur throughout the stratigraphic sequence, the majority of ichthyosaur remains are found in the shales of the *H*. *serpentinum* zone^11^. Specimen SSN8DOR11 was collected from a temporarily exposed limestone unit (‘Unterer Stein’) belonging to the lower part (εII_5_) of the *H*. *serpentinum* zone in the municipality of Dotternhausen. Notably, ichthyosaur fossils are rare in this bed compared to the shales above and underneath it^11^.

**Part B. Specimen details and taxonomic assignment**

As preserved, the incomplete flipper (SSN8DOR11) has a total length of 99.5 cm, including the extensive soft tissues that encapsulate the individual digits in their natural position; the maximum width is 20.3 cm. Five digits are present, including what are probably four primary ones (II–V)^12^ and a postaxial accessory digit (Fig. 1 and Extended Data Fig. 1). Although the radiale, distal carpal 2, metacarpal II, and practically all phalanges occur in near-perfect articulation, parts of the proximal limb are missing, including the humerus, epipodials (except for a possible section of the radius) and the remaining carpals and metacarpals. Furthermore, two proximal elements of digit IV are dislocated and lay isolated in the matrix posterior to the articulated portion of the fin.

Digit II is the most complete finger and comprises a total of 19 articulated elements. Almost all of these are notched, although the proximalmost one—which we identify as the radiale based on its shape and size—seemingly lacks this feature (the fragmentary anterior margin of this bone precludes a confident determination as to whether notching is present or not). Similarly, the distalmost phalanx does not bear a notch, whereas phalanx 13 only shows an incipient indentation. Phalanx 10 is malformed, likely because of its close association with a small supernumerary bone.

Digit III comprises a consecutive series of 13 articulated elements, and we identify the proximalmost bone as the first phalanx. This digit is markedly shorter than digit II and includes a very small distal phalanx. In addition, phalanx 8 shows an anomalous accessory ossification with a smaller supernumerary bone.

Digit IV is the shortest of all digits. Notwithstanding the two displaced elements (Fig. 1), this digit includes six consecutive bones, of which we identify the proximalmost one as phalanx 5.

Digit V comprises 13 preserved elements and extends distally further than any other finger. The first preserved phalanx is possibly the eighth one, although this is difficult to confirm given the incomplete nature of the flipper. Similar to digit III, the distalmost phalanx is exceedingly small.

The postaxial accessory digit contains nine successive elements, with the first one being positioned approximately half of the estimated original length of the flipper. The last four elements are very small, similar to the distalmost phalanges of the previous digits.

Six ichthyosaur genera are recognised from the Posidonia Shale, including *Temnodontosaurus*, *Eurhinosaurus*, *Suevoleviathan*, *Stenopterygius*, *Hauffiopteryx*, and *Magnipterygius*^13–15^. Through detailed comparisons with the fore- and hindlimbs of these taxa, SSN8DOR11 can be confidently identified as a front flipper of *Temnodontosaurus*. This genus is known from at least seven species, although a thorough assessment is required to determine how many of these remain valid^16–19^. In the Posidonia Shale, only one species is currently accepted: *T. trigonodon*; notably, this was the first ichthyosaur to be formally named from the formation^20^. Based on the following character state combination, SSN8DOR11 can be reliably assigned to *T. trigonodon*: (1) the presence of at least three, but probably four primary digits; (2) the extent of notching in digit II; (3) the number of elements in digit II; (4) the reduced length of digit IV compared to the other fingers; (5) the great length of digit V; (6) the sub-angular morphology of the proximal elements with a transition into very small, rounded phalanges; and (7), to a lesser extent, the overall size of the flipper^12,13,17^.

Several specimens of *T. trigonodon* are known—including practically intact and articulated skeletons—which primarily derive from the vicinity of the town of Holzmaden in south-western Germany. One of the most complete of these fossils is SMNS 50000. By direct comparison with SSN8DOR11, the various character states listed above can be readily observed in both specimens (Fig. 1 and Extended Data Fig. 3a), although there are some notable differences. In SMNS 50000, the elements in the distalmost part of each digit are more compact than in SSN8DOR11; however, this is likely due to taphonomic and/or ontogenetic differences, as the front flipper of the former fossil is approximately 20% larger than the latter (see further details below). Comparing what we identify as digit V, the shape, curvature and distal extent are similar, although the extremity does not reach as far in SMNS 50000 as it does in SSN8DOR11. This variation could reflect individual differences, but may also be artificial, e.g., the tiny distal elements remain buried within the matrix, did not preserve in the first place, or were mistakenly removed during the preparation of SMNS 50000. Likewise, while it appears that the proximalmost part of digit V could be lacking in SSN8DOR11, a comparison with the virtually identical forefin in UMH-0020 (another articulated skeleton of *T*. *trigonodon* comprising a prepared skull but purposely unprepared postcrania, including an unrestored front flipper; see Extended Data Fig. 3b, c) indicates that this digit is probably complete or missing only one or two proximal elements.

Regarding the identification of digits, there are some discrepancies in the literature as to whether *Temnodontosaurus* has three or four primary digits, and whether digit V should instead be considered an accessory one^12,13,17^. In a detailed study of ichthyosaur forefins (that we follow), Motani^12^ stated that digit V shows truncated development in *Temnodontosaurus*, although he importantly noted that this finger could be better developed in specimens other than those he had access to. It is further worth mentioning that postaxial accessories are variably present in specimens of the same genus, e.g., in *Stenopterygius*, *Ichthyosaurus* and *Protoichthyosaurus*^12,21–23^. Moreover, although the arrangement and number of elements in the mesopodium is invariant in Early Jurassic ichthyosaur genera, the distal flipper morphology can vary between individuals of the same species, and sometimes even within the same specimen^22^.

Most previously recorded flippers of *T. trigonodon* show some degree of adduction; notably, though, considerable variation on this theme exists: whereas a few specimens have closely spaced fingers (e.g., SMNS 50000), others (e.g., SMNS 52340) exhibit digits that are set more widely apart. This discrepancy could be due to ontogenetic differences; however, given that similarly merged digits occasionally can be seen also in other ichthyosaur taxa (extremities devoid of soft tissues seem to be particularly prone to contraction, compare Extended Data Fig. 3d, e), we are more inclined to consider this phenomenon to be the result of taphonomic processes acting on the decomposing limbs. Adducting digits is further at odds with the finger configuration of extant aquatic tetrapods, which all show parallel to splayed out poses (e.g., ref. 24, figs. 1, 6).

While resting on the seafloor, decaying organic matter can attach to the underlying substrate, thereby maintaining skeletal fidelity by limiting current-induced movements of the bones on the downward-directed side of vertebrate carcasses^25^. Moreover, a taphonomic scenario that is broadly comparable to that we envisage for parvipelvian flippers has been previously proposed for extinct aquatic turtles^26,27^. In these fossils, the digits are often adducted and additionally lack adhering soft-tissue traces, a preservational mode thought to reflect an absence of stiffening scaly skin when the animals were alive^26,27^. Curiously, all hitherto documented *Temnodontosaurus* flippers are represented solely by skeletal remains. Thus, it is plausible that SSN8DOR11 is the first fossil in which the digits remain in life position.

**Part C. Ontogenetic assessment**

*Temnodontosaurus* is the largest ichthyosaur of the Early Jurassic, and initiated the scientific study of the group more than 200 years ago^13,28,29^. Although species of this genus vary in size, *Temnodontosaurus* is known from multiple individuals with estimated overall lengths of 10 m or more^13,19,28,29^. Specimens with skull and/or jaw lengths exceeding one metre are generally considered to be skeletally mature^13^; however, little research has focused on the ontogenetic development of this genus and the species contained within it. This is largely due to the rarity of small-sized examples that can be readily assigned to a specific species, with only some fossils bearing enough characters to be confidently identified, including a juvenile *T*. *platyodon* from near Lyme Regis in southern England (ref. 30; see also ref. 31).

SSN8DOR11 represents a skeletally immature (subadult) individual. As stated above, the total length of the flipper (excluding the missing humerus and radius) is about one metre, including the extended fleshy tip. If we omit the soft tissues and instead measure the limb from the first preserved bone (the radiale) to the distalmost element (in digit V), we end up with a length of approximately 73 cm (~79 cm, if an incipient distal ossification is included). When accounting for the missing radius and humerus (but excluding the soft tissues), this gives an estimated total skeletal flipper length of just under a metre, although this does incorporate the diminutive distal elements of digit V that could have been missed or not preserved in larger individuals. One of these is SMNS 50000, a nearly complete and articulated skeleton that measures approximately 9 m in length. This specimen preserves an intact (left) forefin, which has an overall skeletal length (including the humerus) of 116 cm (Extended Data Fig. 3a). Interestingly, this is roughly 20% longer than the estimated original skeletal length of SSN8DOR11. Moreover, the isolated flipper clearly derives from a smaller (and thus presumably younger) individual because the diminutive, oval to discoidal distal phalanges in each of the five digits are spaced more widely apart than in SMNS 50000 (compare Fig. 1 and Extended Data Fig. 3a), and additionally contain substantial amounts of calcified cartilage (Extended Data Fig. 7h, i); features that also characterise immature cetaceans (Extended Data Fig. 7a–c, f, g). Taking all of this into consideration, it is reasonable to assume that the individual to which SSN8DOR11 belonged probably had a body length of between 6.5 and 7.5 m.

It is finally worth noting that specimen UMH-0020 has a total skeletal fin length of about 75 cm (including the humerus), when measured to the miniscule distal elements of digit V. This flipper is thus shorter than SSN8DOR11, but otherwise provides a good comparison because of the very similar extent of digit V (Extended Data Fig. 3b). Although a complete body length cannot be measured for UMH-0020, this fossil has a skull length of 106 cm, which indicates that the skull was around 1.2 m in the individual yielding SSN8DOR11.

**Part D. Osteoderms**

Osteoderms are bone-rich structures that reinforce the integument in select members of most tetrapod lineages^32,33^. Aside from being both morphologically diverse and capable of attaining a broad range of sizes, these skeletal elements all share the following trait combination: (1) they have an origin within the dermis; (2) are composed primarily of osseous tissue; and (3) develop without true cartilaginous preformation^32^. The only possible exceptions pertain to the presence of chondroid bone (a calcified tissue that is distinct from, yet morphologically intermediate between, cartilage and bone)^34^ in developing osteoderms of some animals^35^, and placodonts—seagoing reptiles of the Triassic Period^36^—which reportedly possess ‘fibro-cartilaginous bone’ as part of their protective dermal armour^37^.

Chondroid bone (CB) cells can resemble chondrocytes^38^, and small regions with a CB-like matrix have been identified in, e.g., osteoderms of the American alligator, *Alligator mississippiensis*^35^. However, despite some histomorphological similarities, cartilage is lacking in these integumental ossifications, to suggest bone formation without a pre-existing cartilage scaffold^39^. Moreover, CB lacks the prominent globular organisation typical of the ichthyosaur dermal cartilage documented herein.

Likewise, the chondrocyte lacunae-like microstructures in placodont osteoderms^37^ have alternatively been explained as voids left behind by decayed large-diameter collagen fibre bundles^32^. Regardless of interpretation, the placodont plate tissue is both structurally and histologically different from the globular calcified cartilage we identify in *Temnodontosaurus* (e.g., it lacks well-defined isogenous cell clusters, Liesegang banding patterns, and morphologically distinct territorial and interterritorial matrices).

Notably, whereas hupehsuchians—Triassic aquatic reptiles that are phylogenetically close to ichthyopterygians^40,41^—exhibit one or more layers of ‘dermal ossicles’ (osteoderms *sensu* ref. 32) above the vertebral column (and occasionally an additional row along the gastralia)^42^, ichthyosaurs are considered to lack such integumentary mineralisations^43,44^.

**Part E. Reconstructing the front flipper of *Temnodontosaurus***

The skeletal diagram of the reconstructed flipper in Figure 4 is based on SSN8DOR11, with missing elements taken from SMNS 15950, 17560, 17980, 50000, and UMH-0020 (all referable to *Temnodontosaurus trigonodon* and size-adjusted before being added to the illustration). The extent of soft tissue along the trailing edge was estimated by approximation of dimensional data and comparisons with well-preserved forelimbs of the genera *Ichthyosaurus* (NHMUK R1664, Natural History Museum, London, UK) and *Stenopterygius* (e.g., GPIT-PV-30017; MH 686, Urwelt-Museum Hauff, Holzmaden, Germany; PMU 24321 and 24323, Museum of Evolution Palaeontology, Uppsala, Sweden; and SMNS 7401, 7800 and 81960). In these parvipelvians, the tissue depth does not exceed 60% of the corresponding depth of the skeletal support, and additionally remains relatively constant along the fin span. The precise location of the detached soft-tissue patch (Fig. 1 and Extended Data Fig. 2) is unknown, but must be somewhere in the proximo-posterior region of the extremity—an assumption that is corroborated by the inferred original position of the associated phalanx.

We also calculated the aspect ratio (AR) of our reconstructed forefin (4.85) using an equation previously employed by, e.g., Woodward *et al*.^45^ to determine the ARs of extant whale flippers:

AR = Length^2^/Planform Area

where ‘Length’ (which is identical to span) is the distance from the distal humeral epiphysis (our definition) to the fleshy flipper tip, and ‘Planform Area’ the planar surface area of the fin blade. This same equation was further employed to establish the AR of some other ichthyosaur soft-tissue forefins, including GPIT-PV-30017 (1.94), PMU 24321 (2.11), PMU 24323 (1.95), SMNS 7401 (2.06) (all assignable to *Stenopterygius*), and NHMUK OR29672 (4.14) (*Protoichthyosaurus*); notably, because of its incomplete nature (see ref. 46, pl. 20), the latter specimen necessitated extrapolation of both the skeletal and soft parts using the left front flipper of the holotype (UNM.G.2017.1, University of Nottingham Museum, Nottingham, UK) of *P*. *applebyi* as a template (see ref. 22, fig. 6).

**Part F. Notes on analyses and experiments**

**Methodological approach**

Our integrated scientific approach combines a suite of sensitive imaging (e.g., SRXTM, FEG-SEM and TEM), elemental (EDX) and molecular (ToF-SIMS and IR microspectroscopy) techniques (see ‘Methods’), which have previously been employed in tandem to determine the structural and chemical composition of multimillion-year-old animal soft-tissue remains, including eyes^47,48^, skin^10,49–51^, feathers^52^, and internal organs^50^. The methods of choice supplement each other by measuring different properties of the investigated materials; they further allow imaging in both two and three dimensions down to the sub-micrometre level, in addition to providing spatially resolved molecular information that can be linked directly to discrete microstructures in our samples. Furthermore, in order to gain as much knowledge as possible about the constituent organic and inorganic matter, as well as to establish the degree of taphonomic influence, both untreated and demineralised samples from SSN8DOR11 were examined. Ground sections were further produced to analyse the chondroderm and bone histology, whereas our harbour porpoise (*Phocoena phocoena*) samples were used in a comparative investigation aimed primarily at studying the relationship between soft tissues and skeletal support in an extant flipper-hydrofoil. The porpoise samples also served as a ‘hands-on’ complement to our recent previous investigations^50,51^ and the published literature^53^ on the structural make-up of amniote integument. Finally, CFD simulations were conducted to explore the hydrodynamic and acoustic effects of passive flow control devices on a virtual section of SSN8DOR11.

**Time-of-flight secondary ion mass spectrometry**

Positive- and negative-ion ToF-SIMS spectra obtained from untreated SSN8DOR11 epidermis evinced a predominance of fluorine-containing calcium phosphate, with minor contributions from calcium carbonate. In addition, organic ions were localised to irregular patches of varying dimensions (measuring from a few to ~30 µm in diameter) embedded within the inorganic matrix (Extended Data Fig. 5a, b). The shape, size and distribution of these carbonaceous ‘spots’ were consistent with aggregations of melanosome-like microbodies observed under FEG-SEM (Extended Data Fig. 4f), to suggest a melanophore origin^50^—an interpretation that was further strengthened by negative-ion ToF-SIMS data acquired from the microstructures, revealing spectral congruence with a *Sepia officinalis* eumelanin reference sample (Extended Data Fig. 5c).

More extensive organic structures (up to ~100 µm in diameter) were also observed on the fossil surface, corresponding to exposed portions of the underlying melanosome layer that feasibly represents the juxtaposed epidermal–dermal interface and superficial dermis. Positive- and negative-ion ToF-SIMS spectra derived from a demineralised sample of this accumulation were dominated by carbonaceous materials. In negative ion mode, the fossilised surface revealed spectral similarities with a *Sepia* eumelanin reference sample, although the relative intensity of nitrogen-containing ions was considerably lower (Extended Data Fig. 5d). A comparable intensity reduction of nitrogen-containing ions has been previously documented also in other fossils, and attributed to partial degradation of the eumelanin molecular structure^10^. This interpretation is here further corroborated by specific features occurring in positive-ion spectra that are characteristic of polyaromatic hydrocarbons (PAHs)^54^, which are lacking in the eumelanin reference spectrum (Extended Data Fig. 5e). Collectively, our ToF-SIMS data indicate the presence of eumelanin pigment residues that are incompletely diagenetically transformed into PAH-like compounds.

FEG-SEM micrographs of the demineralised skin layer exhibited a largely flat surface, albeit with random circular features measuring about 20 µm in diameter (Extended Data Fig. 5f). A high-resolution ToF-SIMS image of the same area revealed a predominance of organic materials, with a patchy distribution of silica as the only other component (notably, no spatial correlation could be observed between the silica and topographic features). At higher magnification, FEG-SEM imaging showed that the surface mainly comprised densely packed microbodies (Extended Data Fig. 5g) with shapes and sizes consistent with previously described remnant melanosomes^10,49,50^.

ToF-SIMS analysis of ‘cellular’ and ‘extracellular’ matter released from demineralised chondroderms exhibited two types of inorganic particles intertwined with a predominantly organic matrix (Extended Data Fig. 6e–h). While the liberated chondrocyte-like bodies were composed of either calcium phosphate or a mixture of zinc (Zn), silver (Ag), sulfur (S), and chlorine (Cl), the fibrous to vesicular organic matter was dominated by PAHs (Extended Data Fig. 6e–h and Table S1). The Zn/Ag/S/Cl-containing material was identified by a suite of ions with equivalent spatial distributions (Table S1), and corroborated by a close match between measured and theoretical isotope patterns. Rare carbonaceous ‘chondrocytes’ were additionally observed under FEG-SEM (Fig. 3l), and their chemical composition determined by EDX; however, no comparable microstructures could be identified during the course of our ToF-SIMS investigation.

Table S1. Selection of positive and negative ions (ToF-SIMS data) representing PAHs and the two types of ‘cellular bodies’ (Ca_x_PO_y_ and Zn/Ag/S/Cl) retrieved from demineralised chondroderms (Extended Data Fig. 6e–h).

|  | Negative ion | *m/z* |  | Positive ion | *m/z* |
| --- | --- | --- | --- | --- | --- |
| PAH | C_4_H^–^ | 49 |  | C_8_H_9_^+^ | 105 |
|  | C_6_H^–^ | 73 |  | C_9_H_7_^+^ | 115 |
|  | C_8_H^–^ | 97 |  | C_10_H_8_^+^ | 128 |
|  | C_10_H^–^ | 121 |  | C_11_H_9_^+^ | 139 |
|  | C_12_H^–^ | 145 |  | C_12_H_8_^+^ | 152 |
|  |  |  |  | C_13_H_9_^+^ | 165 |
|  |  |  |  | C_14_H_10_^+^ | 178 |
|  |  |  |  | C_15_H_9_^+^ | 189 |
|  |  |  |  | C_16_H_10_^+^ | 202 |
|  |  |  |  | C_17_H_11_^+^ | 215 |
|  |  |  |  | C_18_H_10_^+^ | 226 |
|  |  |  |  | C_19_H_11_^+^ | 239 |
| Ca_x_PO_y_ | PO_2_^–^ | 63 |  | CaPO_2_^+^ | 103 |
|  | PO_3_^–^ | 79 |  | Ca_2_O_2_H^+^ | 113 |
|  |  |  |  | Ca_2_OF^+^ | 115 |
|  |  |  |  | Ca_2_PO_3_^+^ | 159 |
|  |  |  |  | Ca_2_PO_4_^+^ | 175 |
|  |  |  |  | Ca_3_PO_5_^+^ | 231 |
| Zn/Ag/S/Cl | S^–^ | 32 |  | Zn^+^ | 64 |
|  | Cl^–^ | 35, 37* |  | Ag^+^ | 107, 109* |
|  | ZnSCl^–^ | 131, 133, 135* |  | Ag_3_S^+^ | 353, 355, 357, |
|  | ZnCl_3_^–^ | 169, 171, 173* |  |  | 359* |
|  | AgCl_2_^–^ | 177, 179, 181* |  |  |  |

*Multiple *m/z* values for the same ion indicate major isotopes.

**Computational fluid dynamics**

## ***Noise attenuation by passive flow control devices***

Noise is pressure fluctuations that travel with the speed of sound^55,56^. Because vortices in a flow induce pressure fluctuations, they also create noise. The frequency of the sound generated by vortices depends on their size: large vortices produce low-frequency sounds that propagate over long distances, whereas small vortices lead to high-frequency noise that is dampened faster^55–57^. Passive flow control devices introduce small disturbances into the flow, which enhance the breakup of larger vortices, in turn causing noise attenuation^58^. Therefore, there is an expected reduction of the noise amplitude in the lower frequency regime that usually is accompanied by a slight increase at higher frequencies^58^. Nevertheless, because higher frequency sounds are dampened faster in water^59^, the use of noise abatement structures still is beneficial for most animals^57,58,60^.

An undesirable side-effect of passive flow control devices can be a reduction in hydrodynamic performance. Hence, in an optimal configuration, any device should disturb the flow sufficiently to trigger a breakdown of larger vortices, while concurrently having a negligible impact on the hydrodynamic forces acting on the body^58^.

### ***Constructing the flipper geometry***

Models of multiscale fluid flows and their interactions with biological structures provide valuable information about the core functions of specific features^61–63^. Accordingly, we performed computational fluid dynamics simulations to numerically investigate potential hydroacoustic benefits of trailing edge serrations and surface treatments on a virtual section of SSN8DOR11 (Fig. 5 and Extended Data Figs. 9, 10), without any *a priori* assumption regarding the nature of the emitted noise (e.g., tonal or broadband). In our digital replica (Fig. 5a), the chord length and serration size, as well as the width and spacing of the ornamentations, were based on direct measurements from the flipper at ~75% of the span; however, because the original thickness and cross-sectional outline of the extremity could not be determined from the flattened fossil, the hydrofoil profile was instead constructed from dimensional data obtained from the forelimb of the living minke whale, *Balaenoptera acutorostrata*^64^. This cetacean was chosen as a reasonable modern analogue because its flippers are elongate and somewhat ‘wing-like’, yet dorsoventrally rather narrow, and thus broadly comparable to those of *Temnodontosaurus* (assuming the existence of a relationship between phalanx thickness and overall fin depth in this ichthyosaur that was not completely overprinted by the encasing connective tissues). Notably, the resulting geometry was similar to a NACA 0018 aerofoil, having a maximum thickness of 0.182 chord lengths (c) at 0.305 of the chord.

By necessity, the adopted flipper geometry had to include some simplifications, which in part were motivated by a need to keep the required computing power within reasonable limits. However, other approximations were introduced to isolate the effects of specific parameters. One of the latter pertained to the fin’s length, which was reduced to 0.096 m (~0.5 c). Calculations dealing with a complete flipper would require an immense amount of computing power (especially when considering the large number of analysed cases), but importantly, would not contribute with any noteworthy additional information about vortices generated in the separation region. As a result of these limitations, our virtual model did not consider any influence from tip vortices or input from the adjacent body. Regardless, our primary aim was to study the effect(s) of trailing edge serrations and surface treatments, and these could be readily identified and isolated when the above mentioned body parts were omitted from the design.

At the other end of the spectrum, a computational domain size that is too small inevitably impedes the three-dimensional evolution of vortical structures. Hence, the length of the adopted flipper section included 12 serrations; these were sufficient to describe the evolution of small-scale (that is, order of magnitude of the serrations or less) vortices in the trailing edge region. Notably, we also performed a sensitivity analysis that included a fin geometry that was twice as long (that is, 24 instead of 12 serrations); however, this extension did not substantially change the flow.

The serrations were created by attaching a sine wave of 4 mm amplitude and 8 mm period along the trailing edge of our hydrofoil, and scaling the last 25% of the flipper cross-section (corresponding to 5 cm) to follow the sinusoidal margin (a necessity to achieve an even transition from the gently curved anterior surface to serrated trailing edge). The ridges and troughs both had 0.8 × 0.8 mm rectangular cross-sections and a spacing of 2 mm (as in SSN8DOR11). The height of both surface treatments increased linearly from 0 to 0.8 mm between 5 and 10% of the chord, and decreased again to 0 mm between 90 and 95% of the chord (Extended Data Fig. 9c, d).

## ***Numerical set-up***

The computational domain’s length and height were set to 6 and 2.8 m, respectively (an increase of the domain length and height by 50% did not alter the flow field). The width of the domain matched the adopted flipper length. In terms of chord lengths, the domain size was 30 × 14 × 0.48 c. The model was placed 2 m from the inlet at half height of the domain, and then rotated to achieve the desired angle of attack (α). At the inlet, a steady velocity with a magnitude of 1.5 m/s was introduced^65–67^, whereas at the outlet, zero-gradient conditions were applied to all variables except pressure, which had a fixed value. In addition, eventual backflow into the computational domain was avoided by zeroing the velocity magnitude. The flipper surface was treated as a no-slip wall, whereas slip conditions were imposed at the top and bottom boundaries. For the sides of the computational domain, two different boundary conditions were tested: cyclic arbitrary mesh interface (AMI) and symmetry. AMI induced spurious oscillations; therefore, symmetry was used in all computations. The adopted velocity led to a Reynolds number of 300,000 and a Mach number of 0.001.

An adequate mesh is crucial to guarantee the quality of computational fluid dynamics simulations. Therefore, a mesh sensitivity study was carried out for a serrated geometry at 0° α that featured chordwise ridges across the surface. Three mesh resolutions were considered, comprising approximately 5, 10 and 30 million cells. A comparison of the results along select monitoring lines revealed that the average velocity profiles were more-or-less superimposed onto one another for the three cases. The velocity fluctuations were, however, expected to be more sensitive to the mesh resolution. Indeed, peak fluctuations increased from 0.1 m^2^/s^2^ to 0.16 m^2^/s^2^ (60%) when the mesh was refined from 5 to 10 million cells, whereas at the finest resolution (30 million cells), there was an additional increase to 0.18 m^2^/s^2^ (12.5% compared to the 10 million cell case). Note that when employing large eddy simulations (LES), small differences are expected in comparisons of mesh resolutions, especially for turbulent fluctuations because the cell size used for discretisation has a direct impact on the amount of resolved turbulent kinetic energy. Since the 30 million cell case would require a substantial amount of computing resources, the 10 million mesh was deemed to be sufficient. This conclusion was further supported by the normalised wall distance (y+), with values close to unity along the greater part of the flipper surface (Extended Data Figs. 9a, b, 10a, b).

Time-dependent simulations must be computed in two stages. At the first stage, the flow develops from user-supplied, potentially unphysical initial conditions to a statistically steady flow that fulfils the Navier–Stokes equations. Next, the flow is computed in time to calculate statistics. The data collected at multiple monitoring points indicated that for the present set-up, three seconds (22.5 convection times) were needed to develop the flow and an additional nine seconds (67.5 convection times) to collect the statistical data.

## ***Impact of trailing edge serrations***

The impact of trailing edge serrations was studied by comparing cases with and without these passive flow control devices at five different α (0, 5, 10, 15, and 20°, respectively). Because noise generation is strongly related to the formation and development of vortices, our primary focus was directed towards instantaneous visualisation of the vortex structures and statistics of the velocity fluctuations. Additionally, the emitted sound pressure levels were monitored at multiple locations.

Instantaneous snapshots of vortices (visualised using the Lamda2 method)^68^ produced around the flipper section at 0° α are shown in Extended Data Figure 9 for a geometry without (Extended Data Fig. 9a) and with (Extended Data Fig. 9b) trailing edge serrations. It is noteworthy that the transversal vortices generated in the boundary layer break down and lead to the formation of a turbulent region in the vicinity of the trailing edge. Serrations did not appear to alter (at least not in a visible way) the flow around the fin, not even when examining the velocity fluctuation levels. Consequently, only velocity fluctuations recorded around the serrated fin geometry are shown in Extended Data Figure 9e, f and g. At 0° α, the velocity fluctuation field is symmetric, and the largest fluctuations occur in the shear layer region (Extended Data Fig. 9e). At 5° α, the flow field becomes asymmetric, and the separation point is moved upstream (Extended Data Fig. 9f). The strongest fluctuations occur on the suction side, in the region where the transition to turbulence takes place, which suggests that the separation point is unsteady; that is, it fluctuates back and forth. For the 10° α case, the separation occurs near the leading edge, but the maximum fluctuation levels are lower than in the previous (5° α) case (Extended Data Fig. 9g).

Sound pressure levels recorded by a virtual microphone located 250 m upstream of the flipper section (corresponding to ~36 body lengths) are compared for five serrated and five unserrated cases in Extended Data Figure 9h. At 0° α (black lines), the emitted noise levels are comparable in the frequency range beneath ~60 Hz (Strouhal number, St = 8.0, when based on the inlet velocity magnitude and chord length) for the serrated (solid lines) and unserrated (dashed lines) cases. However, at higher frequencies, a noise reduction of approximately 1–3 dB can be seen. When α is increased to 5° (blue lines), the overall noise levels increase due to a larger separation region, leading to a larger volume of vortices and stronger fluctuation levels (Extended Data Fig. 9f). Interestingly, the largest noise levels recorded in the 5° α case exceed those observed for 10° α between 30 and 60 Hz (St = 4.0–8.0) (Extended Data Fig. 9h). These high levels can be attributed to the strong velocity fluctuations seen on the suction side of the hydrofoil (Extended Data Fig. 9f). At 10° α (green lines), the noise difference between the serrated and unserrated geometry is negligible (Extended Data Fig. 9h). For low frequencies (up to ~100 Hz, St = 13.3), the emitted noise levels are mostly of the same order of magnitude or less than at 5° α, which could be due to the fact that lower frequencies are generated by larger vortices, and that the size of the largest eddies in both the 5 and 10° α cases are of the same order of magnitude. At higher (>110 Hz, St = 14.7) frequencies, the radiated noise levels for the 10° α case exceed those at 5° α. These levels are related to small vortices, and the larger separation region seen for the 10° α case leads to a larger volume of small eddies radiating more acoustic energy. At 15° α (red lines), the noise levels increase considerably at both low (~20 Hz, St = 2.7) and higher (>70 Hz, St = 9.3) frequencies (Extended Data Fig. 9h). The peak observed around 10 Hz (St = 1.3) is attributed to the near-stall regime. The noise emitted by the unserrated (dashed red line) and serrated (continuous red line) geometries are again of the same order of magnitude. For the greatest α considered (20°, magenta lines), noise levels are high over the entire frequency range because of stall. A comparison of sound levels at several upstream microphones revealed an expected decrease in noise with increasing distance from the flipper model (Extended Data Fig. 9i).

## ***Noise abatement by surface treatments***

Instantaneous snapshots of vortices produced around the flipper section are shown in Extended Data Figure 10 for a geometry covered with either ridges (Extended Data Fig. 10a) or troughs (Extended Data Fig. 10b). The generation of small-scale eddies in the vicinity of the surface treatments is readily apparent in both cases. When compared against the case with a smooth surface (Extended Data Fig. 9b), small-scale vortices emerge further upstream; that is, closer to the leading edge. This is reflected also in the velocity statistics. Extended Data Figure 10c–e show average velocity fluctuations for a smooth (but serrated) geometry at 0° α (Extended Data Fig. 10c), as well as for serrated hydrofoils equipped with either ridges (Extended Data Fig. 10d) or troughs (Extended Data Fig. 10e). Velocity fluctuations emerge at approximately 55% of the chord in the smooth flipper case, whereas they appear already at about 35–40% in the presence of surface treatments. It is further noteworthy that the lighter blue colour in the wake (Extended Data Fig. 10d, e) indicates that the velocity fluctuations are smaller in this region when surface treatments are added to our model.

Similar conclusions can be drawn also from the results obtained at 5° α (Extended Data Fig. 10f–h); that is, surface treatments lead to earlier development of vortices and a reduction of the maximum velocity fluctuation magnitude. It is interesting to note that surface treatments diminish fluctuations even along the suction side (Extended Data Fig. 10f–h). This decrease is attributed to the small eddies generated by the ornamentations that trigger a faster breakdown of the large-scale vortices.

The radiated acoustic noise levels reflect the same phenomena as observed in the velocity fluctuations. At 0° α (Extended Data Fig. 10i), there is a substantial (~5–7 dB) noise reduction in the low-frequency (<50 Hz, St = 6.7) region for a serrated flipper section equipped with either ridges or troughs; the noise attenuation of both surface treatments being of comparable magnitude. For higher frequencies (>80 Hz, St = 10.7), the geometries with surface treatments emit more noise than the smooth one (in addition, ridges generate more noise than troughs). The increased noise levels can be attributed to small-scale vortices generated near the fin surface by these ornamentations. When α is increased to 5° (Extended Data Fig. 10j), the noise reduction in the low-frequency regime is even more extreme (up to ~10 dB) than at 0° α. It is also accompanied by a minor decrease in the frequency interval below ~130 Hz (St = 17.3). At higher (>140 Hz, St = 18.7) frequencies, the noise levels are similar in all considered cases, in part presumably because the small-scale vortices have a comparable amount of energy. Extended Data Figure 10i and j further reveal that with increasing upstream distance, an expected noise reduction occurs; however, no noteworthy qualitative change is apparent, and thus the damping or amplification of the noise levels caused by surface treatments is similar irrespective of distance.

***Combined effect of passive flow control devices***

Our computations revealed that trailing edge serrations can reduce noise over a range of low (<200 Hz, St = 26.7) frequencies (Fig. 5e), whereas ridges and troughs dampen the emitted noise primarily in the frequency region below ~70 Hz (St = 9.3) (Fig. 5f). Hence, the combined effect of these passive flow control devices implies a noise reduction potential over a span of low frequencies. The depicted plots (Fig. 5 and Extended Data Figs. 9, 10) describe radiated noise upstream of the flipper model because this is where a prey would be. Nonetheless, a comparable attenuating effect of the emitted noise was recorded also by microphones located in other directions. We focused primarily on frequencies below ~200 Hz (St = 26.7), which was motivated in part by our intention to emphasise those frequency intervals where the impact of the investigated structures changes. Furthermore, the dampening effect on the acoustic noise by the surrounding medium (water) increases at higher frequencies; therefore, these are less relevant for a hunting pelagic animal^59,69^.

**Part G. Notes on the taphonomy**

Previous records of isolated ichthyosaur flippers have been attributed to either predation, scavenging or decay (see ref. 70 and references therein). With its abundant soft-tissue structures, SSN8DOR11 represents an unprecedented *Temnodontosaurus* find; yet, the preservational fidelity and degree of articulation vary substantially between different parts of the fossil. While the bones in the mid–distal portion of the fin remain mostly in life position relative to one another and additionally are surrounded by a continuous sheet of relict integument, the humerus and all skeletal elements in the proximo-posterior part are either displaced or absent. In addition, a patch of soft tissue has been removed from its original position to rest a short distance posterior to the main section of the limb. Given that taphonomic processes often are complex and even can be unpredictable^25^, it is difficult to determine exactly what happened to the flipper prior to it being buried and subsequently fossilised. However, it is reasonable to assume that the fin somehow got dislodged from the rest of the body, perhaps by a predator or scavenger targeting the base of the extremity (notably, a variety of pathologies have been previously documented in specimens of *Temnodontosaurus*, including forefins)^71^. The dismembered appendage then settled on the seabed with its largest area more-or-less parallel to the surface of the substrate, a posture in which most of the skeletal elements ended up being close to, or just below, the water–sediment interface (assuming that the flipper sank some distance into the viscous mud)^72^. It is further possible that the soft-tissue envelope of the semi-buried limb had partially ruptured, leaving the larger bones of the thicker, proximal part temporarily exposed to bottom currents, which caused dislocation and dispersal of some elements^25,73^. The detached skin strip is harder to explain, but could be the result of scavenging (although no obvious bite marks can be found on the tissue piece) before adherence to the sediment^25,73^. The flipper was then completely covered by muddy deposits, and diagenetic compaction later reduced the initially three-dimensional forelimb into a flattened fossil coated on the underside by remnant integument.

The conservation and fossilisation process that followed included permeation of the decaying soft tissues by fluids supersaturated in calcium and phosphate (resulting in a partial replacement of the biological material with calcium phosphate and, at a later stage, void infill by calcium carbonate) and an incomplete transformation of the remaining organics into aliphatic and aromatic geomacromolecules, as previously described for other Posidonia Shale ichthyosaurs^10,50^. Importantly, this combination of authigenic mineralisation and *in situ* polymerisation resulted in the retention of three types of organs and tissues: skin, bone and calcified cartilage.

The presumably dark-coloured integument likely was mineralised from the exterior and inwards, leading to a melange of cellular preservation and replication by calcium phosphate in the outer epidermis (Fig. 2d and Extended Data Figs. 4f, 5a–c), and more advanced degradation of the innermost epidermis and superficial dermis into a condensed mat of densely aggregated melanosomes (Fig. 2g and Extended Data Fig. 5d–g)^50^. However, in similarity with many other soft-tissue fossils^50,74–77^, dermal and/or underlying connective tissues were selectively phosphatised (Fig. 2c, h, i and Extended Data Fig. 4e, i, k), perhaps owing to the mineral-binding ability and nucleative properties of the constituent fibrous matter^50,78–80^.

Whereas non-biomineralised cartilage normally does not survive across deep time, naturally mineralised (calcified) cartilage is well-documented in the fossil record^81–86^. This preferential preservation of calcified over unmineralised cartilage is apparent also in SSN8DOR11: while the cartilage caps of the bones are retained in pristine condition, the presumably unmineralised interphalangeal cartilage^87^ is lacking (Extended Data Fig. 7). The chondroderms were also mineralised *in vivo*, as demonstrated by recurring differences in the density of phosphatisation between regions immediately surrounding the chondrocyte lacunae (the territorial matrix) and the (interterritorial) matrix in between these ‘cell nests’ (Fig. 3h and Supplementary Video 2) (see ref. 84 for details). Enrichment of fluorine (as evinced by our FEG-SEM/EDX and ToF-SIMS analyses) further indicates that some compositional modifications occurred *post mortem*^88^. Similarly, although a few ‘cellular bodies’ remain predominantly carbonaceous (Fig. 3l), the vast majority of the sub-spherical microstructures liberated by our acid treatment are preserved either as a zinc/silver chloride/sulphide mixture or in calcium phosphate (Fig. 3m, Extended Data Fig. 6e–h and Supplementary Video 1), to suggest secondary infill of the empty chondrocyte lacunae during diagenesis.

**Part H. Function of the flippers in *Temnodontosaurus***

When an organism travels through water, the component particles of the fluid are set into motion, causing the emission of pressure waves in all directions^57,60^. Such oscillatory movements include sound waves plus other hydrodynamic forces that can be perceived by receivers with special flow-sensing capabilities^57,60^. Because both acoustic and mechanosensory cues provide biologically meaningful information about the environment, aquatic animals possess different types of particle motion detectors (such as the auditory and lateral line systems of fish) to identify and localise incoming hydrodynamic signals^57,60^. Sensitive hearing and/or mechanosensory organs are of particular importance for organisms in habitats where light is scarce, or when the ecological niche requires them to hunt either at night or under turbid conditions^57^.

When approaching prey, a predator disrupts fluid, creating perturbations in the flow field ahead of it^60,89^. Likewise, swimming motions produce low to mid-range frequency noise, particularly during speed bursts and turning manoeuvres^90^. Because these signals can be intercepted by the intended target and trigger an unwanted escape response, hunting animals that do not wish to give their presence away have evolved ways to reduce perceivable water movements^60,89^. In addition, special noise suppression devices may exist in some living marine mammals^58,90^. For example, the flippers of the humpback whale, *Megaptera novaeangliae*, are exceptional among cetaceans in that they are equipped with sinusoidally arranged tubercles along the leading edge. These protuberances act as passive flow-regulating structures, contributing not only to enhanced hydrodynamic performance but presumably also to dampen self-generated noise as the limbs slice through the water^90^. Furthermore, the propulsive fluke carries a jagged trailing edge, comparable in certain respects to the fringed remiges (wing feathers) that promote silent flight in owls^58^.

Due to the vibrant nature of the marine soundscape, predatory animals that either search for or pursue prey use various means and strategies to avoid being detected. For instance, killer whales (*Orcinus orca*) live in cohesive social groups, and like most odontocetes, normally rely on acoustic signaling for underwater communication^91^. However, when hunting for other marine mammals (which are known to have a good sense of hearing), they go silent^92^, a condition some researchers^93^ consider to be a ‘stealth’ mode. Nonetheless, although highly efficient predators in their own right, neither orcas nor any other extant seagoing amniote (save perhaps for the sperm whale, *Physeter macrocephalus*) are as specialised for scotopic conditions as was *Temnodontosaurus*. Not only does this large-bodied parvipelvian have the largest eyeballs of any vertebrate (see below), but it likely also possessed flow control devices to diminish trailing edge self-noise (and potentially plankton bioluminescence triggered by the moving animal)^94^, to suggest that it was uniquely suited for life in dim-lit pelagic environments and thus without any directly comparable modern ecological analogue.

As outlined above, structures thought to reduce the acoustic signature of living animals are rare, and mostly confined to body parts (e.g., wings of owls and fluke of the humpback whale) that oscillate during locomotion^58,90^. Hence, we conservatively predict that the thrust-producing tail fin of *Temnodontosaurus* (which currently is known only from skeletal remains) originally carried noise suppression devices that were at least as elaborate as those herein documented in the front flippers. Moreover, even if examples from nature are scarce, there is no shortage of reports dealing with trailing edge serrations and surface treatments from physics and engineering points of views^58,95–101^. Collectively, these studies provide compelling numerical and experimental evidence for the effectiveness of such devices in attenuating noise emitted from both aero- and hydrofoils. In addition, certain geometries have been shown to simultaneously reduce drag^97,99^, whereas others are accompanied with a slight aero/hydrodynamic penalty^96,98^. Notably, the impact on the hydrodynamic performance by the inclusion of trailing edge serrations and surface treatments to our digital model is minimal, at least for the considered α (Fig. 5b, c).

While we hypothesise that the elongate forefins of *Temnodontosaurus* primarily provided enhanced hydrodynamic lift at low cruising speeds (this ichthyosaur may occasionally even have drifted with ocean currents as do certain long-finned pelagic sharks today)^102,103^, they likely also served other, non-mutually exclusive functions (e.g., to furnish stability and trim). The extant humpback whale has greatly enlarged (25–33% of body length) and highly mobile flippers to generate the force necessary for ‘acrobatic’ lunging manoeuvres^104–106^. While the forefins of *Temnodontosaurus* certainly were used for, e.g., steering and pitch changes (and even may have been capable of active stroke-generation)^107,108^, they potentially could have likewise permitted tight turning manoeuvres. However, contrary to humpback whale flippers, which are equipped with prominent tubercles (sinusoidal outgrowths) as a means to delay stall and improve the hydrodynamic performance at high α^104,105,109^, leading edge devices are lacking in the proportionally shorter (~14–17% of estimated body length) extremities of *Temnodontosaurus*, to suggest that they instead were adapted for functions other than increased agility. Similarly, although some surface treatments have been shown to affect the flow separation dynamics at the stall phase^110,111^, our computational simulations indicate that the ridges/troughs in SSN8DOR11 likely were too small to have any impact on the stall characteristics of the forefin.

Although stealth likely was pivotal when foraging, *Temnodontosaurus* presumably used a variety of strategies to overtake and catch elusive prey. For instance, its body was relatively elongate by parvipelvian standards, and thus must have been rather bendable^108^. In similarity with extant dolphins^112^, this flexibility could have enabled *Temnodontosaurus* to intercept and secure cephalopods and other small-sized animals by improved turning performance of the head and mouth.

**Part I. Large eyes and adaptations for stealth in ichthyosaurs**

Bigger eyes always allow for better visual performance (acuity, contrast sensitivity and/or visual speed), especially in dim-light conditions^94^. However, this relationship follows the law of diminishing returns, which states that the larger an eye is, the less it pays off to further increase it in size^94^. This is the reason why eyes, even in very large animals, normally do not exceed ~10 cm in diameter. The only known exceptions to this tenet are extant cephalopods of the genera *Architeuthis* and *Mesocychoteuthis*, and parvipelvian ichthyosaurs, whose eyeballs can grow up to three times the size of those of other animals of comparable body length^113,114^. In living giant and colossal squids, this enlargement likely is a consequence of an evolutionary arms race between the visual range of these coleoids and sonar range of their main predator, the sperm whale^114,115^. Because both *Architeuthis* and *Mesocychoteuthis* inhabit environments below the photic zone, their eyes are uniquely suited for spotting plankton bioluminescence triggered by approaching whales^114,115^. Thus, under these special conditions, the balance between performance and cost has been pushed towards massive eyes in giant and colossal squids, and advanced sonar in the sperm whale (large eyeballs and loud sonar are expensive to build and maintain, but importantly increase the detection range well beyond that of other animals).

It is conceivable that a similar arms race between predator and prey resulted in the exceptional visual and stealth adaptations seen in *Temnodontosaurus*. In this instance, however, the ichthyosaur is the predator^116–119^, and it is disputed whether or not it engaged in deep diving^71,120,121^. Hence, we conservatively infer that *Temnodontosaurus* inhabited the epipelagic zone and, possibly also, uppermost layer of the mesopelagic zone, where it could rely on downwelling light for visual prey detection. Moreover, given the enormous proportions of its eyes, light availability must have been limited, to suggest that *Temnodontosaurus* typically hunted either at depths where sunlight was faint and/or at night or dusk/dawn. We further hypothesise that silent swimming (stealth) emerged as a means to avoid being detected by prey equipped with coevolving hearing and/or tactile senses.

**Part J. References cited in the Supplementary Information**

1. Röhl, H.-J., Schmid-Röhl, A., Oschmann, W., Frimmel, A. & Schwark, L. The Posidonia Shale (Lower Toarcian) of SW-Germany: an oxygen-depleted ecosystem controlled by sea level and palaeoclimate. *Palaeogeogr*., *Palaeoclim*., *Palaeoecol*. **165**, 27–52 (2001).
2. Röhl, H.-J. & Schmid-Röhl, A. Lower Toarcian (Upper Liassic) black shales of the Central European Epicontinental Basin: a sequence stratigraphic case study from the SW German Posidonia Shale. *SEPM Spec*. *Publ*. **82**, 165–189 (2005).
3. Galasso, F., Schmid-Röhl, A., Feist-Burkhardt, S., Bernasconi, S. M. & Schneebeli-Hermann, E. Changes in organic matter composition during the Toarcian Oceanic Anoxic Event (T-OAE) in the Posidonia Shale Formation from Dormettingen (SW-Germany). *Palaeogeogr*., *Palaeoclim*., *Palaeoecol*. **569**, 110327 (2021).
4. Muscente, A. D. et al. What role does anoxia play in exceptional fossil preservation? Lessons from the taphonomy of the Posidonia Shale (Germany). *Earth-Sci*. *Rev*. **238**, 104323 (2023).
5. Williams, M., Benton, M. J. & Ross, A. The Strawberry Bank Lagerstätte reveals insights into Early Jurassic life. *J*. *Geol*. *Soc*. **172**, 683–692 (2015).
6. Sinha, S. et al. Global controls on phosphatization of fossils during the Toarcian Oceanic Anoxic Event. *Sci*. *Rep*. **11**, 24087 (2021).
7. Frimmel, A., Oschmann, W. & Schwark, L. Chemostratigraphy of the Posidonia Black Shale, SW Germany: I. Influence of sea-level variation on organic facies evolution. *Chem*. *Geol*. **206**, 199–230 (2004).
8. Dickson, A. J. et al. Molybdenum-isotope chemostratigraphy and paleoceanography of the Toarcian Oceanic Anoxic Event (Early Jurassic). *Paleoceanography* **32**, 813–829 (2017).
9. Them, T. R. et al. Thallium isotopes reveal protracted anoxia during the Toarcian (Early Jurassic) associated with volcanism, carbon burial, and mass extinction. *Proc*. *Natl* *Acad*. *Sci*. **115**, 6596–6601 (2018).
10. De La Garza, R. G., Sjövall, P., Hauff, R. & Lindgren, J. Preservational modes of some ichthyosaur soft tissues (Reptilia, Ichthyopterygia) from the Jurassic Posidonia Shale of Germany. *Palaeontology* **66**, e12668 (2023).
11. Maxwell, E. E. & Vincent, P. Effects of the early Toarcian Oceanic Anoxic Event on ichthyosaur body size and faunal composition in the Southwest German Basin. *Paleobiology* **42**, 117–126 (2016).
12. Motani, R. On the evolution and homologies of ichthyopterygian forefins. *J*. *Vert*. *Paleontol*. **19**, 28–41 (1999).
13. McGowan, C. & Motani, R. Ichthyopterygia. In *Handbook of Paleoherpetology* (ed. Sues, H.-D.) Vol. 8, 175 pp. (Verlag Dr. Friedrich Pfeil, Munich, 2003).
14. Maxwell, E. E. New metrics to differentiate species of *Stenopterygius* (Reptilia: Ichthyosauria) from the Lower Jurassic of southwestern Germany. *J*. *Paleontol*. **86**, 105–115 (2012).
15. Maisch, M. W. & Matzke, A. T. *Magnipterygius huenei* n. gen. n. sp., a new small stenopterygiid (Reptilia: Ichthyosauria) from the Posidonienschiefer Formation of SW Germany. *N. JB. Geol. Palaönt. Abh*. **303**, 169–201 (2022).
16. Maisch, M. W. Phylogeny, systematics, and origin of the Ichthyosauria – the state of the art. *Palaeodiversity* **3**, 151–214 (2010).
17. Swaby, E. J. & Lomax, D. R. A revision of *Temnodontosaurus crassimanus* (Reptilia: Ichthyosauria) from the Lower Jurassic (Toarcian) of Whitby, Yorkshire, UK. *Hist*. *Biol*. **33**, 2715–2731 (2020).
18. Laboury, A., Bennion, R. F., Thuy, B., Weis, R. & Fischer, V. Anatomy and phylogenetic relationships of *Temnodontosaurus zetlandicus* (Reptilia: Ichthyosauria). *Zool*. *J*. *Linn*. *Soc*. **195**, 172–194 (2022).
19. Larkin, N. R. et al. Excavating the ‘Rutland Sea Dragon’: the largest ichthyosaur skeleton ever found in the UK (Whitby Mudstone Formation, Toarcian, Lower Jurassic). *Proc*. *Geol*. *Ass*. **134**, 627–640 (2023).
20. Theodori, C. Über einen kolossalen *Ichthyosaurus trigonodon*. *Gelehrte Anzeigen der Königlich Bayerischen Akademie der Wissenschaften, München* **16**, 906–911 (1843).
21. Caldwell, M. W. Limb ossification patterns of the ichthyosaur *Stenopterygius*, and a discussion of the proximal tarsal row of ichthyosaurs and other neodiapsid reptiles. *Zool*. *J*. *Linn*. *Soc*. **120**, 1–25 (2008).
22. Lomax, D. R., Massare, J. A. & Mistry, R. T. The taxonomic utility of forefin morphology in Lower Jurassic ichthyosaurs: *Protoichthyosaurus* and *Ichthyosaurus*. *J*. *Vert*. *Paleontol*. **37**, e1361433 (2017).
23. Massare, J. A. & Lomax, D. R. A taxonomic reassessment of *Ichthyosaurus communis* and *I. intermedius* and a revised diagnosis for the genus. *J. Syst. Palaeontol*. **16**, 263–277 (2018).
24. DeBlois, M. C. & Motani, R. Flipper bone distribution reveals flexible trailing edge in underwater flying marine tetrapods. *J*. *Morph*. **280**, 908–924 (2019).
25. Orr, P. J. et al. “Stick ‘n’ peel”: explaining unusual patterns of disarticulation and loss of completeness in fossil vertebrates. *Palaeogeogr*., *Palaeoclim*., *Palaeoecol*. **457**, 380–388 (2016).
26. Joyce, W. G., Mäuser, M. & Evers, S. W. Two turtles with soft tissue preservation from the platy limestones of Germany provide evidence for marine flipper adaptations in Late Jurassic thalassochelydians. *PLoS ONE* **16**, e0252355 (2021).
27. Augustin, F. J. et al. A new specimen of *Solnhofia parsonsi* from the Upper Jurassic (Kimmeridgian) Plattenkalk deposits of Painten (Bavaria, Germany) and comments on the relationship between limb taphonomy and habitat ecology in fossil turtles. *PLoS ONE* **18**, e0287936 (2023).
28. McGowan, C. Giant ichthyosaurs of the Early Jurassic. *Can*. *J*. *Earth Sci*. **33**, 1011–1021 (1996).
29. Ji, C. et al. Phylogeny of the Ichthyopterygia incorporating recent discoveries from South China. *J*. *Vert*. *Paleontol*. **36**, e1025956 (2016).
30. McGowan, C. *Temnodontosaurus risor* is a juvenile of *T. platyodon* (Reptilia: Ichthyosauria). *J. Vert. Paleontol*. **14**, 472–479 (1995).
31. Massare, J. A., Lomax, D. R., Williams, M. & Howells, C. A catalog of the Lower Lias ichthyosaurs in the Charles Moore collection. *Paludicola* **14**, 141–193 (2024).
32. Vickaryous, M. K. & Sire, J.-Y. The integumentary skeleton of tetrapods: origin, evolution, and development. *J*. *Anat*. **214**, 441–464 (2009).
33. Williams, C. et al. A review of the osteoderms of lizards (Reptilia: Squamata). *Biol*. *Rev*. **97**, 1–19 (2021).
34. de Buffrénil, V. & Quilhac, A. in *Vertebrate Skeletal Histology and Paleohistology* (eds de Buffrénil, V. et al.) 147–182 (CRC Press, Boca Raton, 2021).
35. Dubansky, B. H. & Dubansky, B. D. Natural development of dermal ectopic bone in the American alligator (*Alligator mississippiensis*) resembles heterotopic ossification disorders in humans. *Anat*. *Rec*. **301**, 56–76 (2018).
36. Scheyer, T. M. & Klein, N. in *Vertebrate Skeletal Histology and Paleohistology* (eds de Buffrénil, V. et al.) 425–434 (CRC Press, Boca Raton, 2021).
37. Scheyer, T. M. Skeletal histology of the dermal armor of Placodontia: the occurrence of ‘postcranial fibro-cartilaginous bone’ and its developmental implications. *J*. *Anat*. **211**, 737–753 (2007).
38. Klein, N. & Scheyer, T. M. Vertebral, rib, and osteoderm morphology and histology of Middle Triassic diapsid *Eusaurosphargis*. *Acta Palaeontol*. *Pol*. **69**, 633–648 (2024).
39. Dubansky, B., Raney, J. & Dubansky, B. Mode of ossification and extracellular fiber characterization of osteoderm matrix in the American alligator (*Alligator mississippiensis*) and comparisons to inherited and acquired heterotopic ossification disorders. *FASEB J*. **34**, 1 (2020).
40. Carroll, R. L. & Zhi-Ming, D. *Hupehsuchus*, an enigmatic aquatic reptile from the Triassic of China, and the problem of establishing relationships. *Phil*. *Trans*. *R*. *Soc*. *Lond*. *B* **331**, 131–153 (1991).
41. Chen, X.-H., Motani, R., Cheng, L., Jiang, D.-Y. & Rieppel, O. The enigmatic marine reptile *Nanchangosaurus* from the Lower Triassic of Hubei, China and the phylogenetic affinities of Hupehsuchia. *PLoS ONE* **9**, e102361 (2014).
42. Wu, X.-C., Zhao, L.-J., Sato, T., Gu, S.-X. & Jin, X.-S. A new specimen of *Hupehsuchus nanchangensis* Young, 1972 (Diapsida, Hupehsuchia) from the Triassic of Hubei, China. *Hist*. *Biol*. **28**, 43–52 (2016).
43. Scheyer, T. M., Oberli, U., Klein, N. & Furrer, H. A large osteoderm-bearing rib from the Upper Triassic Kössen Formation (Norian/Rhaetian) of eastern Switzerland. *Swiss J*. *Palaeontol*. **141**, 1 (2022).
44. Ebel, R., Herrel, A., Scheyer, T. M. & Keogh, J. S. Review of osteoderm function and future research directions. *J*. *Zool*. **325**, 1–24 (2025).
45. Woodward, B. L., Winn, J. P. & Fish, F. E. Morphological specializations of baleen whales associated with hydrodynamic performance and ecological niche. *J*. *Morph*. **267**, 1284–1294 (2006).
46. Owen, R. XIX.—A description of some of the soft parts, with the integument, of the hind-fin of the *Ichthyosaurus*, indicating the shape of the fin when recent. *Trans*. *Geol*. *Soc*. *Lond*. **6**, 199–201 (1841).
47. Lindgren, J. et al. Molecular preservation of the pigment melanin in fossil melanosomes. *Nat*. *Commun*. **3**, 824 (2012).
48. Lindgren, J. et al. Interpreting melanin-based coloration through deep time: a critical review. *Proc*. *R*. *Soc*. *B* **282**, 20150614 (2015).
49. Lindgren, J. et al. Skin pigmentation provides evidence of convergent melanism in extinct marine reptiles. *Nature* **506**, 484–488 (2014).
50. Lindgren, J. et al. Soft-tissue evidence for homeothermy and crypsis in a Jurassic ichthyosaur. *Nature* **564**, 359–365 (2018).
51. De La Garza, R. G. et al. An ancestral hard-shelled sea turtle with a mosaic of soft skin and scutes. *Sci*. *Rep*. **12**, 22655 (2022).
52. Lindgren, J. et al. Molecular composition and ultrastructure of Jurassic paravian feathers. *Sci*. *Rep*. **5**, 13520 (2015).
53. Landmann, L. in *Biology of the Integument* (eds Bereiter-Hahn, J. et al.) 150–187 (Springer, Berlin, 1986).
54. Stephan, T., Jessberger, E. K., Heiss, C. H. & Rost, D. TOF-SIMS analysis of polycyclic aromatic hydrocarbons in Allan Hills 84001. *Met*. *Planet*. *Sci*. **38**, 109–116 (2003).
55. Michelsen, A. in *Bioacoustics: a Comparative Approach* (ed. Lewis, B.) 3–38 (Academic Press, London, 1983).
56. Doolan, C. & Moreau, D. *Flow Noise Theory* (Springer Nature Singapore, Singapore, 2022).
57. Hawkins, A. D. & Myrberg, A. A. in *Bioacoustics: a Comparative Approach* (ed. Lewis, B.) 347–405 (Academic Press, London, 1983).
58. Wolfe, T. M. Review of fluid dynamic and acoustic performance of biologically inspired passive flow control trailing edge devices for design applications. *55^th^ AIAA Aerospace Sciences Meeting*, *AIAA SciTech Forum*, AIAA 2017-0542 (2017).
59. Tyack, P. L. & Janik, V. M. in *Animal Communication and Noise* (ed. Brumm, H.) 251–271 (Springer, Berlin Heidelberg, 2013).
60. Hanke, W. in *Flow Sensing in Air and Water—Behavioral, Neural and Engineering Principles of Operation* (eds Bleckmann, H. et al.) 3–29 (Springer, Heidelberg, 2014).
61. Watts, P. & Fish, F. E. The influence of passive, leading edge tubercles on wing performance. *Proc*. *Twelfth Intl*. *Symp*. *Unmanned Untethered Submers*. *Technol*. Durham New Hampshire: Auton. Undersea Syst. Inst. (2001).
62. Fish, F. E. & Lauder, G. V. Passive and active flow control by swimming fishes and mammals. *Annu*. *Rev*. *Fluid Mech*. **38**, 193–224 (2006).
63. Zhang, Z., Wang, Q. & Zhang, S. Review of computational fluid dynamics analysis in biomimetic applications for underwater vehicles. *Biomimetics* **9**, 79 (2024).
64. Cooper, L. N. et al. Hydrodynamic performance of the minke whale (*Balaenoptera acutorostrata*) flipper. *J*. *Exp*. *Biol*. **211**, 1859–1867 (2008).
65. Motani, R. Scaling effects in caudal fin propulsion and the speed of ichthyosaurs. *Nature* **415**, 309–312 (2002).
66. Motani, R. Swimming speed estimation of extinct marine reptiles: energetic approach revisited. *Paleobiology* **28**, 251–262 (2002).
67. Fish, F. E. in *Convergent Evolution—Animal Form and Function* (eds Bels, V. L. & Russell, A. P.) 477–522 (Springer, Cham, 2023).
68. Jeong, J. & Hussain, F. On the identification of a vortex. *J*. *Fluid Mech*. **285**, 69–94 (1995).
69. Ladich, F. in *Animal Communication and Noise* (ed. Brumm, H.) 65–90 (Springer, Berlin Heidelberg, 2013).
70. Stinnesbeck, W. et al. A Lower Cretaceous ichthyosaur graveyard in deep marine slope channel deposits at Torres del Paine National Park, southern Chile. *Geol*. *Soc*. *Am*. *Bull*. **126**, 1317–1339 (2014).
71. Pardo-Pérez, J. M. et al. Pathological survey on *Temnodontosaurus* from the Early Jurassic of southern Germany. *PLoS ONE* **13**, e0204951 (2018).
72. Martill, D. M. Soupy substrates: a medium for the exceptional preservation of ichthyosaurs of the Posidonia Shale (Lower Jurassic) of Germany. *Kaupia* **2**, 77–97 (1993).
73. Reisdorf, A. G. et al. Reply to “Ichthyosaur embryos outside the mother body: not due to carcass explosion but to carcass implosion” by van Loon (2013). *Palaeobio*. *Palaeoenv*. **94**, 487–494 (2014).
74. Lingham-Soliar, T. A unique cross section through the skin of the dinosaur *Psittacosaurus* from China showing a complex fibre architecture. *Proc*. *R*. *Soc*. *B* **275**, 775–780 (2008).
75. Lingham-Soliar, T. & Wesley-Smith, J. First investigation of the collagen *D*-band ultrastructure in fossilized vertebrate integument. *Proc*. *R*. *Soc*. *B* **275**, 2207–2212 (2008).
76. Lindgren, J., Everhart, M. J. & Caldwell, M. W. Three-dimensionally preserved integument reveals hydrodynamic adaptations in the extinct marine lizard *Ectenosaurus* (Reptilia, Mosasauridae). *PLoS ONE* **6**, e27343 (2011).
77. Lindgren, J., Kaddumi, H. F. & Polcyn, M. J. Soft tissue preservation in a fossil marine lizard with a bilobed tail fin. *Nat*. *Commun*. **4**, 2423 (2013).
78. Weldon, P. J., Flachsbarth, B. & Schulz, S. Natural products from the integument of nonavian reptiles. *Nat*. *Prod*. *Rep*. **25**, 738–756 (2008).
79. Balakrishnan, S. et al. Studies on calcification efficacy of stingray fish skin collagen for possible use as scaffold for bone regeneration. *Tissue Eng*. *Regen*. *Med*. **12**, 98–106 (2015).
80. Zhang, W. et al. Biomimetic intrafibrillar mineralization of type I collagen with intermediate precursors-loaded mesoporous carriers. *Sci*. *Rep*. **5**, 11199 (2015).
81. Coates, M. I., Sequeira, S. E. K., Sansom, I. J. & Smith, M. M. Spines and tissues of ancient sharks. *Nature* **396**, 729–730 (1998).
82. Janvier, P. & Arsenault, M. Calcification of early vertebrate cartilage. *Nature* **417**, 609 (2002).
83. Donoghue, P. C. J., Sansom, I. J. & Downs, J. P. Early evolution of vertebrate skeletal tissues and cellular interactions, and the canalization of skeletal development. *J*. *Exp*. *Zool*. (*Mol*., *Dev*., *Evol*.) **306B**, 278–294 (2006).
84. Johanson, Z., Kearsley, A., den Blaauwen, J., Newman, M. & Smith, M. M. No bones about it: an enigmatic Devonian fossil reveals a new skeletal framework—a potential role of loss of gene regulation. *Sem*. *Cell Develop*. *Biol*. **21**, 414–423 (2010).
85. Lemierre, A. & Germain, D. A new mineralized tissue in the early vertebrate *Astraspis*. *J*. *Anat*. **235**, 1105–1113 (2019).
86. Quilhac, A. in *Vertebrate Skeletal Histology and Paleohistology* (eds de Buffrénil, V. et al.) 123–138 (CRC Press, Boca Raton, 2021).
87. Bindellini, G., Wolniewicz, A. S., Miedema, F., Dal Sasso, C. & Scheyer, T. M. Postcranial anatomy of *Besanosaurus leptorhynchus* (Reptilia: Ichthyosauria) from the Middle Triassic Besano Formation of Monte San Giorgio (Italy/Switzerland), with implications for reconstructing the swimming styles of Triassic ichthyosaurs. *Swiss J*. *Palaeontol*. **143**, 32 (2024).
88. Keenan, S. W. From bone to fossil: a review of the diagenesis of bioapatite. *Am*. *Min*. **101**, 1943–1951 (2016).
89. Gemmell, B. J., Adhikari, D. & Longmire, E. K. Volumetric quantification of fluid flow reveals fish’s use of hydrodynamic stealth to capture evasive prey. *J*. *R*. *Soc*. *Interface* **11**, 20130880 (2014).
90. Fish, F. E. Advantages of aquatic animals as models for bio-inspired drones over present AUV technology. *Bioinspir*. *Biomim*. **15**, 025001 (2020).
91. Thomsen, F., Franck, D. & Ford, J. K. B. On the communicative significance of whistles in wild killer whales (*Orcinus orca*). *Naturwissenschaften* **89**, 404–407 (2002).
92. Riesch, R. & Deecke, V. B. Whistle communication in mammal-eating killer whales (*Orcinus orca*): further evidence for acoustic divergence between ecotypes. *Behav*. *Ecol*. *Sociobiol*. **65**, 1377–1387 (2011).
93. Reeves, R. R., Berger, J. & Clapham, P. J. in *Whales, Whaling, and Ocean Ecosystems* (eds Estes, J. A. et al.) 174–187 (Univ. California Press, Berkeley, 2006).
94. Nilsson, D.-E., Warrant, E. & Johnsen, S. Computational visual ecology in the pelagic realm. *Phil*. *Trans*. *R*. *Soc*. *B* **369**, 20130038 (2014).
95. Arce León, C., Ragni, D., Pröbsting, S., Scarano, F. & Madsen, J. Flow topology and acoustic emissions of trailing edge serrations at incidence. *Exp*. *Fluids* **57**, 91 (2016).
96. Clark, I. A. et al. Bioinspired trailing-edge noise control. *AIAA J*. **55**, 740–754 (2017).
97. Muhammad, C. & Chong, T. P. Mitigation of turbulent noise sources by riblets. *J*. *Sound Vib*. **541**, 117302 (2022).
98. Fiscaletti, D., Luesutthiviboon, S., Avallone, F. & Casalino, D. Streamwise fences for the reduction of trailing-edge noise in a NACA633018 airfoil. *AIAA Pap*. *2022-1925* (2022).
99. Smith, T. A. & Klettner, C. A. Airfoil trailing-edge noise and drag reduction at a moderate Reynolds number using wavy geometries. *Phys*. *Fluids* **34**, 117107 (2022).
100. Hu, Y.-S. et al. Effects of trailing-edge serration shape on airfoil noise reduction with zero incidence angle. *Phys*. *Fluids* **34**, 105108 (2022).
101. Ananthan, V. B. & Akkermans, R. A. D. Trailing edge noise reduction using bio-inspired finlets. *J*. *Sound Vib*. **549**, 117553 (2023).
102. Moss, S. A. *Sharks—an Introduction for the Amateur Naturalist* (Prentice-Hall, Inc., Englewood Cliffs, 1984).
103. Compagno, L. J. V. *Sharks of the World. An Annotated and Illustrated Catalogue of Shark Species Known to Date. Volume 2. Bullhead, Mackerel and Carpet Sharks (Heterodontiformes, Lamniformes and Orectolobiformes)*. FAO Special Catalogue for Fishery Purposes. No. 1, Vol. 2 (FAO, Rome, 2001).
104. Fish, F. E. & Battle, J. M. Hydrodynamic design of the humpback whale flipper. *J*. *Morph*. **225**, 51–60 (1995).
105. Fish, F. E., Weber, P. W., Murray, M. M. & Howle, L. E. Marine applications of the biomimetic humpback whale flipper. *Mar*. *Tech*. *Soc*. *J*. **45**, 198–207 (2011).
106. Segre, P. S., Seakamela, S. M., Meÿer, M. A., Findlay, K. P. & Goldbogen, J. A. A hydrodynamically active flipper-stroke in humpback whales. *Curr*. *Biol*. **27**, R636–R637 (2017).
107. Riess, J. Fortbewegunsweise, Schwimmbiophysik und Phylogenie der Ichthyosaurier. *Palaeontographica Abt*. *A Paläozool. Stratigr.* **192**, 93–155 (1986).
108. Buchholtz, E. A. Swimming styles in Jurassic ichthyosaurs. *J*. *Vert*. *Paleontol*. **21**, 61–73 (2001).
109. Fish, F. E., Weber, P. W., Murray, M. M. & Howle, L. E. The tubercles on humpback whales’ flippers: application of bio-inspired technology. *Integr*. *Comp*. *Biol*. **51**, 203–213 (2011).
110. Yadav, R. & Bodavula, A. Effect of spanwise groove on the dynamic stall characteristics of an airfoil. *J*. *Aerosp*. *Eng*. **236**, 2523–2543 (2022).
111. Raatan, V. S., Ramaswami, S., Mano, S. & Nadaraja Pillai, S. Effect of stall delay characteristics of symmetrical aerofoil using lateral circular ridges. *Wind Str*. **34**, 385–394 (2022).
112. Maresh, J. L., Fish, F. E., Nowacek, D. P., Nowacek, S. M. & Wells, R. S. High performance turning capabilities during foraging by bottlenose dolphins (*Tursiops truncatus*). *Mar*. *Mamm*. *Sci*. **20**, 498–509 (2004).
113. Motani, R., Rothschild, B. M. & Wahl, W. Large eyeballs in diving ichthyosaurs. *Nature* **402**, 747 (1999).
114. Nilsson, D.-E., Warrant, E. J., Johnsen, S., Hanlon, R. & Shashar, N. A unique advantage for giant eyes in giant squid. *Curr*. *Biol*. **22**, 683–688 (2012).
115. Nilsson, D.-E., Warrant, E. J., Johnsen, S., Hanlon, R. T. & Shashar, N. The giant eyes of giant squid are indeed unexpectedly large, but not if used for spotting sperm whales. *BMC* *Evol*. *Biol*. **13**, 187 (2013).
116. McGowan, C. A revision of the longipinnate ichthyosaurs of the Lower Jurassic of England, with descriptions of two new species (Reptilia: Ichthyosauria). *Life Sci*. *Contr*., *R*. *Ont*. *Mus*. **97**, 1–37 (1974).
117. Massare, J. A. Tooth morphology and prey preference of Mesozoic marine reptiles. *J*. *Vert*. *Paleontol*. **7**, 121–137 (1987).
118. Böttcher, R. Über die Nahrung eines *Leptopterygius* (Ichthyosauria, Reptilia) as dem süddeutschen Posidonienschiefer (Unterer Jura) mit Bemerkungen über den Magen der Ichthyosaurier. *Stuttgarter* *Beitr*. *Naturk*. *B* **155**, 1–19 (1989).
119. Serafini, G., Miedema, F., Schweigert, G. & Maxwell, E. E. *Temnodontosaurus* bromalites from the Lower Jurassic of Germany: hunting, digestive taphonomy and prey preferences in a macropredatory ichthyosaur. *Pap*. *Palaeontol*. **11**, e70018 (2025).
120. Fernández, M. S., Archuby, F., Talevi, M. & Ebner, R. Ichthyosaurian eyes: paleobiological information content in the sclerotic ring of *Caypullisaurus* (Ichthyosauria, Ophthalmosauria). *J*. *Vert*. *Paleontol*. **25**, 330–337 (2005).
121. Motani, R. et al. Absence of suction feeding ichthyosaurs and its implications for Triassic mesopelagic paleoecology. *PLoS ONE* **8**, e66075 (2013).
