# Supplementary material for: Adaptations for stealth in the wing-like flippers of a large ichthyosaur
Source: Nature. 2025 Jul 16;644(8078):976–83. doi: 10.1038/s41586-025-09271-w (PMC12390834; doi:10.1038/s41586-025-09271-w)
Supplement: Supplementary file 2 — Reporting Summary [file 41586_2025_9271_MOESM2_ESM.pdf]

nature portfolio

Corresponding author(s): Johan Lindgren

Last updated by author(s): May 27, 2025

Reporting Summary

Nature Portfolio wishes to improve the reproducibility of the work that we publish. This form provides structure for consistency and transparency in reporting. For further information on Nature Portfolio policies, see our Editorial Policies and the Editorial Policy Checklist.

Statistics

For all statistical analyses, confirm that the following items are present in the figure legend, table legend, main text, or Methods section.

n/a	Confirmed	
☐	☒	The exact sample size (*n*) for each experimental group/condition, given as a discrete number and unit of measurement
☐	☒	A statement on whether measurements were taken from distinct samples or whether the same sample was measured repeatedly
☒	☐	The statistical test(s) used AND whether they are one- or two-sided<br>*Only common tests should be described solely by name; describe more complex techniques in the Methods section.*
☒	☐	A description of all covariates tested
☒	☐	A description of any assumptions or corrections, such as tests of normality and adjustment for multiple comparisons
☐	☒	A full description of the statistical parameters including central tendency (e.g. means) or other basic estimates (e.g. regression coefficient) AND variation (e.g. standard deviation) or associated estimates of uncertainty (e.g. confidence intervals)
☒	☐	For null hypothesis testing, the test statistic (e.g. *F*, *t*, *r*) with confidence intervals, effect sizes, degrees of freedom and *P* value noted<br>*Give P values as exact values whenever suitable.*
☒	☐	For Bayesian analysis, information on the choice of priors and Markov chain Monte Carlo settings
☒	☐	For hierarchical and complex designs, identification of the appropriate level for tests and full reporting of outcomes
☒	☐	Estimates of effect sizes (e.g. Cohen's *d*, Pearson's *r*), indicating how they were calculated

Our web collection on statistics for biologists contains articles on many of the points above.

Software and code

Policy information about availability of computer code

Data collection	Polarised and UV imaging: Adobe Photoshop (v.CC 22.3.0)<br>FEG-SEM/EDX: Aztec, version 3.3 and 6.1 (Oxford Instruments Nanotechnology Tools Ltd.) and Zeiss SmartSEM v6<br>TEM: TEM Centre for JEM1400 Plus software<br>X-ray computed microtomography: XradiaReconstructorApp V11.0<br>ToF-SIMS: SurfaceLab, versions 6.7, 7.1 and 7.3 (IONTOF GmbH, Münster, Germany)<br>IR microspectroscopy: Bruker OPUS 8.5 (Bruker, Ettlingen, Germany), Agilent Resolutions Pro 5.3.0 (Agilent Technologies Inc.)<br>CFD: OpenFOAM v2112, v2206
Data analysis	FEG-SEM/EDX: Aztec, versions 3.3 and 6.1 (Oxford Instruments Nanotechnology Tools Ltd.)<br>X-ray computed microtomography: 3D Slicer 4.6.2 (3D Slicer image computing platform); Drishti 3.0 (VizLab, National Computational Infrastructure, Canberra, Australia)<br>SRXTM: Voxler 3, SAVU 4.2 (doi.org/10.5281/zenodo.6900630), gridrec (doi:10.1107/S0909049512032864), and 3D Slicer 5.2.2<br>ToF-SIMS: SurfaceLab, versions 6.7, 7.1 and 7.3 (IONTOF GmbH, Münster, Germany)<br>IR microspectroscopy: Bruker OPUS 8.5 (Bruker, Ettlingen, Germany), Agilent Resolutions Pro 5.3.0 (Agilent Technologies Inc.) and Quasar 1.7.0<br>CFD pre-processing:<br>- cfMesh, version 1.1.2<br>- Custom code used to generate the flipper geometry (59). (59) is a reference to the Figshare repository accompanying the submission.<br>CFD post-processing:<br>- ParaView 5.7.0<br>- Grace 5.1.25

- Spectra computed using custom code (59) based on the open-source fftw library. (59) is a reference to the Figshare repository accompanying the submission.

For manuscripts utilizing custom algorithms or software that are central to the research but not yet described in published literature, software must be made available to editors and reviewers. We strongly encourage code deposition in a community repository (e.g. GitHub). See the Nature Portfolio guidelines for submitting code & software for further information.

Data

Policy information about availability of data

All manuscripts must include a data availability statement. This statement should provide the following information, where applicable:

- Accession codes, unique identifiers, or web links for publicly available datasets
- A description of any restrictions on data availability
- For clinical datasets or third party data, please ensure that the statement adheres to our policy

All data required for assessing the conclusions are contained in the Article, Extended Data Figures 1–10 and Supplementary Information. The raw X-ray computed microtomographic and SRXTM data (Fig. 3f, h and Supplementary Videos 1, 2) are provided in the accompanying MorphoSource (60) repository.
(60) is a reference to the MorphoSource repository accompanying the submission.

Research involving human participants, their data, or biological material

Policy information about studies with human participants or human data. See also policy information about sex, gender (identity/presentation), and sexual orientation and race, ethnicity and racism.

Reporting on sex and gender	N/A
Reporting on race, ethnicity, or other socially relevant groupings	N/A
Population characteristics	N/A
Recruitment	N/A
Ethics oversight	N/A

Note that full information on the approval of the study protocol must also be provided in the manuscript.

Field-specific reporting

Please select the one below that is the best fit for your research. If you are not sure, read the appropriate sections before making your selection.

☒ Life sciences ☐ Behavioural & social sciences ☐ Ecological, evolutionary & environmental sciences

For a reference copy of the document with all sections, see nature.com/documents/nr-reporting-summary-flat.pdf

Life sciences study design

All studies must disclose on these points even when the disclosure is negative.

Sample size	The sample size is limited to a single fossil specimen, SSN8DOR11 (Temnodontosaurus).
Data exclusions	No data were excluded from the analyses.
Replication	Five chondroderm, three skin, five bone/ossifications, and three cartilage samples were collected from SSN8DOR11 and its associated matrix; 12 of these were demineralised. All samples were photographically documented, together with comparative tissues from extant vertebrates. The following experiments were repeated independently with similar results: LM (histological sections): SSN8DOR11 five samples, Phocoena three samples; FEG-SEM: SSN8DOR11 15 samples; EDX: SSN8DOR11 10 samples; TEM: SSN8DOR11 four samples; X-ray computed microtomography: two sets of scans from two chondroderms; SRXTM: six sets of scans from six samples (four chondroderm and two skin samples); IRIS: each pixel in the hyperspectral image in Extended Data Figure 6c consists of 512 co-added individual scans, whereas each pixel in the corresponding image in Extended Data Figure 6d comprises 1,024 co-added individual scans; ToF-SIMS: eumelanin identification, eight measurements from four areas on one sample; eumelanin and PAH identification, three measurements from three areas on one sample; composition of chondrocyte-like bodies, 11 measurements from eight areas on two samples; CFD: the flow developed for 22.5 integral time scales (based on freestream velocity and chord length); statistics were collected for 67.5 integral time scales; the FFTs were computed using 65,536 wide Hanning windows with 50% overlap, resulting in 33 samples. The other experiments, including the X-ray computed tomography, polarised and ultraviolet light imaging and EDX analysis of Phocoena tissues, were not repeated as one set of data from each was deemed as sufficient.
Randomization	Not relevant to this study as no statistical analyses requiring randomization were performed.

Blinding	Not relevant to this study as no analyses requiring blinding were performed.

Reporting for specific materials, systems and methods

We require information from authors about some types of materials, experimental systems and methods used in many studies. Here, indicate whether each material, system or method listed is relevant to your study. If you are not sure if a list item applies to your research, read the appropriate section before selecting a response.

Materials & experimental systems

n/a	Involved in the study
☒	☐ Antibodies
☒	☐ Eukaryotic cell lines
☐	☒ Palaeontology and archaeology
☒	☐ Animals and other organisms
☒	☐ Clinical data
☒	☐ Dual use research of concern
☒	☐ Plants

Methods

n/a	Involved in the study
☒	☐ ChIP-seq
☒	☐ Flow cytometry
☒	☐ MRI-based neuroimaging

Palaeontology and Archaeology

Specimen provenance	SSN8DOR11 was collected by one of us (Georg Göltz) from a temporary exposure of dark, laminated limestone belonging to the Liassic εII5 ('Unterer Stein') part of the Early Toarcian Posidonia Shale in the municipality of Dotternhausen, south-western Germany. The fossil was discovered during construction blasting work, and consequently retrieved as a three-dimensional jigsaw puzzle of odd-sized rock slabs, collectively forming opposing part and counterpart sections of a flipper that has been cleaved along the sagittal plane.
Specimen deposition	The ichthyosaur fossil examined in this study (SSN8DOR11) is permanently accessioned into the collections of Paläontologisches Museum Nierstein, Nierstein, Germany.
Dating methods	Not applicable

☒ Tick this box to confirm that the raw and calibrated dates are available in the paper or in Supplementary Information.

Ethics oversight	This study deals with a single fossil collected from a temporary exposure of the Posidonia Shale in Germany; hence, no ethical approval or guidance was required.

Note that full information on the approval of the study protocol must also be provided in the manuscript.

Plants

Seed stocks	N/A
Novel plant genotypes	N/A
Authentication	N/A
